# A large-scale survey on sharp injuries among hospital-based healthcare workers in China

**DOI:** 10.1038/srep42620

**Published:** 2017-02-16

**Authors:** Xiaodong Gao, Bijie Hu, Yao Suo, Qun Lu, Baiyi Chen, Tieying Hou, Jin’ai Qin, Wenzhi Huang, Zhiyong Zong

**Affiliations:** 1 Department of Infection Control, Zhongshan Hospital, Fudan University, 180 Fenglin Road, Shanghai, China; 2 Department of Infection Control, The Second Affiliated Hospital, Xi’an Jiaotong University, 157 Xiwu Road, Xi’an, Shaanxi,China; 3 Department of Infection Control, The Second Affiliated Hospital, Zhejiang University School of Medicine, 88 Jiefang Road, Hangzhou, Zhejiang, China; 4 Department of Infection Control, The First Hospital, China Medical University, 155 Nanjing Road, Shenyang, Liaoning, China; 5 Department of Infection Control, Guangdong Provincial Hospital, 106 Zhongshan No. 2 Road, Guangzhou, Guangdong, China; 6 Department of Infection Control, The First affiliated Hospital, Guangxi Medical University, 6 Shuangyong Road, Nanning, Guangxi, China; 7 Center of Infectious Diseases and Department of Infection Control, West China Hospital, Sichuan University, Guoxuexiang 37, Chengdu, China

## Abstract

A multi-center survey on sharp injuries (SIs) among hospital-based healthcare workers (HCWs) in seven provinces of China between August and December 2011 was performed. In each province, HCWs from at least 30 hospitals were surveyed by completing a SI report form adapted from the EPINet. The HCWs who declared SIs during the period were interviewed by local infection control practitioners. The survey included 361 hospitals and 206,711 HCWs, most of whom were nurses (47.5%) or doctors (28.4%). In the previous month, 17,506 SI incidents were declared by 13,110 (6.3%) HCWs, corresponding to 1,032 incidents per 1,000 HCWs per year and 121.3 per 100 occupied beds per year. The majority of the SIs was caused by a hollow-bore needle (63.0%). The source patient was identified in 73.4% of all SIs but only 4.4% of all exposures involved a source patient who tested positive for HBV (3.3%), HCV (0.4%) or HIV (0.1%). Only 4.6% of SIs were reported to the infection control team in the hospitals. In conclusion, the rate of SI among HCWs is high in China and SI represents a severe but largely neglected problem. Awareness and safety climate should be promoted to protect the safety of HCWs in China.

Occupational exposure to blood-borne viruses (BBVs) such as hepatitis B virus (HBV), hepatitis C virus (HCV) and human immunodeficiency virus (HIV) is a major threat to healthcare workers (HCWs) during their daily works. The Centers for Disease Control and Prevention (CDC) estimated that about 385,000 percutaneous injuries occurred among HCWs in USA every year^1^. The actual number is likely to be much higher due to widespread underreporting of incidents in USA workplaces^2^. In East Asia, up to 8,319 percutaneous injuries were estimated among HCWs in Taiwan^3^ and a survey on 3,079 registered nurses in 60 hospitals in South Korea revealed that 70.4% of those surveyed declared sharp injuries (SIs)^4^. Occupational exposure can lead to infections of BBVs. It was estimated that about 66,000 HBV, 16,000 HCV and about 1,000 HIV infections might have occurred among HCWs worldwide due to percutaneous injuries in a single year^5^. Therefore, occupational exposure to BBVs resulting from SIs among HCWs is a global problem and represents an often-preventable hazard for HCWs. Preventing SI and subsequent BBV exposure requires a comprehensive approach to prevention and control^6^,^7^.

China has a large population of HCWs. However, unlike developed countries, the rate of SIs among HCWs in China remains largely unknown due to a lack of large-scale occupational incident surveillance programs^5^. A retrospective large-scale multi-site survey on SIs among HCWs was therefore organized and conducted in China.

## Results

A total of 361 hospitals with 173,219 beds in seven provinces (Guangdong, Guangxi, Liaoning, Shaanxi, Shanghai, Sichuan and Zhejiang) participated in the survey. Among 253,555 HCWs who were eligible for the survey, 206,711 responded to the invitation and completed the survey form, corresponding to an 81.5% overall response rate. Most of HCWs surveyed were nurses (47.5%) and doctors (28.4%). The response rate varied significantly among HCWs in different occupational groups with the highest seen in nurses (95.1%) and doctors (88.4%) and the lowest seen in technicians (36.0%; Table 1)

Among the 206,711 HCWs surveyed, 13,110 (6.34%) declared 17,506 SI incidents that occurred in the previous month. The SI rate using the number of HCWs as the denominator was therefore 84.7 incidents per 1,000 HCWs per month, corresponding to 1,032 incidents per 1,000 HCWs per year. The SI rate using the number of occupied beds as the denominator was 10.1 incidents per 100 occupied beds per month, corresponding to 121.3 per 100 occupied beds per year.

Among occupational groups, nurses had the highest SI rate with 7.8% of all nurses surveyed having declared at least one SI in the previous month, corresponding to 103.9 incidents per 1,000 nurses per month and about 1,247 per 1,000 nurse-years (Table 1). Trainees had the second highest SI rate (7.7%), while 5.5% of doctors, 3.8% of logistic workers and 3.2% of technicians declared SIs in the previous month (Table 1).

**Figure 1:**
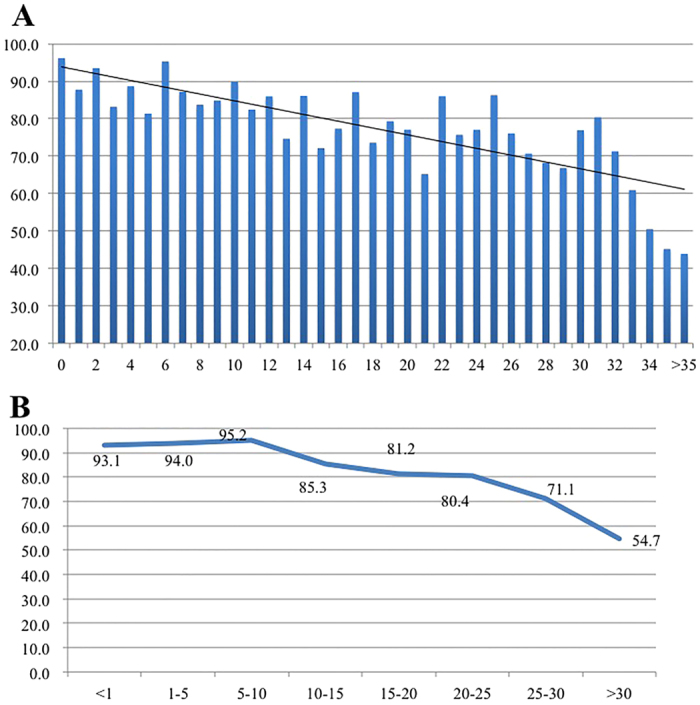
SI rates according to the working years. Panel (A), SI rates according to working years. Panel (B), SI rates according to every ten working years. SI rates are expressed by incidents per 1,000 HCWs per month. Vertical axis is SI rates and horizontal axis is the working years for HCWs. Both trends in Panel A and B were statistically significant (*p* < 0.05).

There was a trend that SI rates among HCWs dropped along with the increased number of working years (Fig. 1 panel A, *p* < 0.05), in particular after 10 years (Fig. 1 panel B, *p* < 0.05). Of note, HCWs who have been working for less than ten years had a higher SI rate than those with more than 10 years working experience (91.3 vs 56.4 incidents per 1,000 HCWs per month, *p* < 0.05).

**Figure 2:**
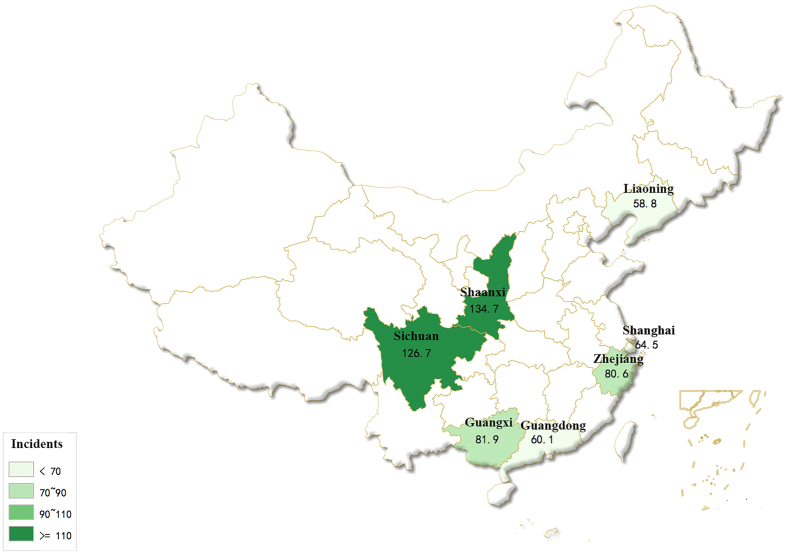
SI rates in the seven provinces. SI rates are expressed as incidents per 1,000 HCWs per month. The map was generated using the free online Chinese map making tool at http://c.dituhui.com/apps with permission.

SI rates, which were not adjusted according to hospital bed capacity, among HCWs in tertiary and secondary hospitals were in general similar (Table 2). Nonetheless, when compared to their counterparts in secondary hospitals, doctors and technicians in tertiary hospitals had slightly higher SI rates. As for different geographic areas, SI rates of HCWs varied from 58.8 incidents per 1,000 HCWs per month in Liaoning to 60.1 in Guangdong, 64.5 in Shanghai, 80.6 in Zhejiang, 81.9 in Guangxi, 126.7 in Sichuan and 134.7 in Shaanxi (Fig. 2).

The location of 16,554 (94.6%) out of 17,506 SI incidents was reported, while data about the location of the remaining SI incidents was not available. Most SIs occurred in patient rooms (42.2%) and operating rooms (21.0%). The common place for the occurrence of SIs varied according to occupational groups (Table 3). For instance, most (61.8%) SIs among doctors occurred in the operating rooms, while more than half (55.4%) among SIs of nurses occurred in the patient rooms.

Most (63.0%) SIs were caused by hollow-bore needles, among which disposable syringe needles (commonly used for withdrawing blood in China) and scalp steel needles (commonly used for intravenous infusion) were the most common sharps causing SIs and accounted for 35.6% and 22.7% of all SIs, respectively. In addition to hollow-bore needles, surgical suture needles were another frequent cause of SIs and accounted for 14.2% of all SIs. Of note, most SIs among doctors were caused by either the surgical suture needles (41.1%) or hollow-bore needles (26.2%), while the majority (76.7%) of SIs for nurses were caused by hollow-bore needles.

Various medical procedures, such as surgical suturing, removing venous infusion needles, recapping needles, dealing with medical waste and preparing fluid infusions (Table 4) led to SIs of HCWs. For doctors, SIs occurred most commonly during surgical suturing (42.9%). For nurses, treating medical wastes (16.8%), removing venous needles (15.5%), preparing fluid infusion (11.8%) and recapping needles (10.7%) were the most common procedures leading to SIs. For logistics workers, dealing with medical wastes led to most (70.8%) SIs.

The source patient was identified in 73.4% (12,856/17,506) of all SIs but most SIs (44.5%) among logistics workers could not be tracked back to a specific source. Among those with the source patient being identified, only 4.4% of all exposures involved a source patient tested positive for a BBV including 3.3% positive for HBV, 0.4% for HCV and 0.1% for HIV.

Although all participating hospitals had the policy that HCWs should report their SIs to the Infection Control Team, only 4.6% of SI incidents had actually been reported when they occurred and underreporting rates of HCWs in different occupational groups were all above 90%, ranging from 92 to 97%. The vast majority (88.9%) of HCWs surveyed declared that they received training to prevent occupational exposures to BBVs. Training appears to make a significant difference as HCWs who received training had a lower SI rate compared to those who did not receive training (6.2% vs 10.3%, *p* < 0.05).

## Discussion

This is the first ever large-scale multi-site survey on SIs among HCWs in China. The major findings of this survey include the following: (1) the SI rate was high (see below for details); (2) nurses and trainees experienced more SIs than other occupational groups such as doctors and technicians; (3) SI rates varied by provinces; (4) most SIs occurred in patient rooms or operating rooms and were caused by hollow-bore needles, especially disposable syringe needles, or by scalpel blades or steel suture needles; (5) the source patient could be identified in most cases of SIs but few source patients were tested positive for a BBV; (6) the vast majority of SIs were not reported; (7) most HCWs declared that they had received training despite high SI rates; (8) training was associated with a lower SI rate.

As mentioned above, the SI rate was estimated as 1,032 incidents per 1,000 HCW-year. In 2011, there were about 3,705,541 HCWs in hospitals in mainland China according to the annual report of the Ministry of Health, China^8^. It could therefore be estimated that about 3.8 million (3,824,118; 1,032 ÷ 1000 × 3,705,541) SIs may have occurred among HCWs in hospitals in mainland China each year. This appears to be much higher than of the figure estimated (384,325 SIs) in USA hospitals each year^9^, although the population of China is about 4 times of that of the USA^10^. The SI rate in mainland China revealed by this survey was also much higher than the 170 incidents per 1,000 HCW-year in Taiwan^11^. However, in the Taiwanese study HCWs were asked to recall SI in the previous year rather than the previous month^11^, which could result in a lower SI rate as some SI episodes which had occurred months ago might not be recalled accurately.

Although there are a number of studies on SIs in the literature, national or region-wide data on SI rates among HCWs are still scarce in the world. Furthermore, comparing nationwide rates is complicated by inconsistency in the methodology to collect and report the numerator, denominator, and the overall rate based on differences in medical resources, available data, and practice. In general, in published studies and reports there are two approaches, either passive (based on actual incident reports from HCWs) or active (based on survey using questionnaires), to collect the number of SI incidents and then to calculate the SI rate or ratio. Using the passive approach, the SI rate per 100 occupied beds was 19.46 in the USA in 2011^12^ and 6.2 in Japan between 2009 and 2011^13^. However, the SI rate based on reported incidents might be significantly lower than the reality due to underreporting. Nonetheless, using questionnaires, the SI rate was 41.8 and 18.0 incidents per 100 occupied beds in Taiwan in 2011^3^ and in 2004–2005^11^, respectively. It is therefore evident that compared to SI rates elsewhere the SI rate among HCWs in mainland China is high.

The exact reasons for the comparatively high SI rate among HCWs in China are not entirely clear but a few factors such as the heavy clinical workload, the lack of the safety culture and rare use of safety devices might have contributed^14^,^15^. There were 3,705,100 hospital beds in mainland China, 107,547,387 patients admitted to hospitals and 1,627,761 registered nurses in hospitals in 2011^8^. Therefore, the nurse-bed ratio was 1:2.3 and the nurse-admission ratio was 1:66.1, which might represent heavy workload for HCWs in China.

Reasons for the variation in SI rates in different provinces also remain unclear but might reflect the effect of training for preventing SIs and the local safety culture. Further investigations are warranted.

Underreporting of SIs among HCWs has been estimated as ranging from 26 to 90% and represents a serious worldwide problem for establishing accurate estimates for risk and burden^16^. In the available multi-site large-scale surveillance studies, underreporting of SIs among HCWs was estimated as 54% in the USA^6^ and 78.8% in Taiwan^11^. Despite the fact that underreporting was also common elsewhere, the 95.4% underreporting rate identified here is higher. We have not specifically investigated the reasons for underreporting and barriers for reporting in this survey but a number of factors may lead to underreporting such as excessive paper work, unfamiliarity with or unawareness of the reporting procedures and negligence of HCWs according to a previous small-scale investigation^17^.

Despite the high SI rate found in this survey the vast majority of HCWs surveyed declared that they had received training to prevent occupational exposures to BBVs. The high SI rate suggests that there is much room for the training to be improved. For instance, training should be targeted at those who work in high-risk areas such as operating rooms, ICUs, and patient rooms where a disproportionate number of SIs occurred^6^.

There are several limitations of this survey. First, recalled SI rates should be interpreted with caution, as recall bias may be present and overall incidents may be inaccurate. Nonetheless, in this survey HCWs were asked to recall SIs in the previous month, a relatively short period, and SIs were therefore more likely to be recalled accurately. Secondly the survey was not designed to investigate the reasons for the common occurrence of SIs, nor was it designed to examine reasons for underreporting or to address preventability of SIs. Thirdly the participating hospitals were not randomly selected. This was due to practicality, as the hospitals were selected by provincial authorities. Nonetheless, as the hospitals were selected arbitrarily from the hospital list in the province and were at various locations of the province, we believe these hospitals could serve as reasonable representatives for hospitals in China. Finally, the survey was not designed to investigate the reasons for the differences in SI incidents by geographic area or hospital or demographic characteristics.

In conclusion, SIs among HCWs are common in China and represent a huge but largely neglected problem. Awareness and actions such as establishing safety climate, improving incident reporting and surveillance programs, and adopting devices with safety features to isolate sharps should be implemented to protect the safety of HCWs in China.

## Methods

This survey was conducted in seven provinces in different parts in China (Fig. 2), i.e. Guangdong and Guangxi in the south, Liaoning in the north, Shaanxi and Sichuan in the west, and Shanghai and Zhejiang in east, between August and December in 2011. In each province, at least 30 hospitals including 15 secondary and 15 tertiary hospitals were selected arbitrarily by the provincial healthcare authorities from the hospital list to represent each province.

Infection control practitioners (ICPs) in each hospital conducted the survey after receiving training in the survey procedures. In this study, HCW refers to a person who works in a hospital and may come into contact with patients and/or their wastes. All HCWs who may come into contact with sharp devices in these hospitals were surveyed as to whether or not they had occupational exposure to blood or body fluids due to SIs in the previous month by completing a report questionnaire, which was collected and interpreted by ICPs. SI refers to a penetrating stab wound from a needle, scalpel, or other sharp object that may result in exposure to blood or other body fluids (http://www.cdc.gov/niosh/stopsticks/sharpsinjuries.html). The questionnaire was translated from the Exposure Prevention Information Network “EPINet” Report for Blood and Body Fluid Exposures and the EPINet Report for Needlestick and Sharp Object Injuries from the International Safety Center (internationalsafetycenter.org) in simplified Chinese and was modified to accommodate the names of clinical departments in China. For those who declared one or more exposures, the detail and potential risk factors of the exposure were obtained by ICPs using an additional follow-up questionnaire to investigate the immune status of HCWs and to track post-exposure test results of HCWs for BBVs. The EPINet Post-Exposure Follow-up Form was used as the follow-up questionnaire here, which was also translated in simplified Chinese. Data from each hospital including the number of HCWs surveyed and the questionnaires for each SI exposure were submitted online via the dedicated site at www.icchina.org.cn/epinet within one week after the survey and were analysed following the EPINet manual^16^.

All HCWs were classified into one of the six occupational groups, i.e. doctors, nurses, technicians who carried out various tests in clinical laboratories, logistic workers who deal with medical wastes, trainees (trainee medical students, nurses or technicians) and others (physical therapists, midwives, anesthetists, medical imaging technicians and cleaners for endoscopy, etc.) who are responsible for patient care and are at risk of BBV exposure.

The survey was approved by the National Institute of Hospital Administration (NIHA), Ministry of Health, China as an evaluation project and was carried out in accordance with the approved guidelines. The participation in the survey was voluntary. Completion of the questionnaires was considered implied consent for survey participation and the informed consent was waived by NIHA.

Statistical analysis was performed using the SPSS program (version 18.0; SPSS Inc., Chicago, IL). Chi square test was used to compare multiple sample rates, to examine the trend of SI rate according to working years and to compare SI rates between tertiary and secondary hospitals. Whether the distribution of the location that SI had occurred and the proportion of medical procedures leading to SIs were statistically significant or not were examined using Fisher’s exact test. The significance level was adjusted to 0.003125 in pairwise comparison of multiple sample rates (Table 1) by using the equation: *α*′ = 0.05/[(k − 1) × k/2 + 1] (k was the number of occupational category); otherwise, a 0.05 *p* value was considered statistically significant.

## Additional Information

**How to cite this article**: Gao, X. *et al*. A large-scale survey on sharp injuries among hospital-based healthcare workers in China. *Sci. Rep.* **7**, 42620; doi: 10.1038/srep42620 (2017).

**Publisher's note:** Springer Nature remains neutral with regard to jurisdictional claims in published maps and institutional affiliations.

## Figures and Tables

**Table 1:** SI incidents and reporting rates among HCWs.

HCW group	Number surveyed	Number of HCW declared SIs	SI incidents	Reported SI incidents	SI rate, % of HCWs^2^	SI rate, 1,000 HCWs per month	SI rate, 100 occupied beds per month	Reporting rate (%)^4^	Source patients being identified, cases (%)
Doctors	58,766	3,223	4,239	170	5.5	72.1	2.4	4.0	3,375 (79.6)
Nurses	98,118	7,642	10,195	446	7.8	103.9	5.9	4.4	7,568 (74.2)
Technicians	9,936	328	418	13	3.3	42.1	0.2	3.1	293 (70.1)
Logistic workers^1^	15,118	575	854	50	3.8	56.5	0.5	5.9	380 (44.5)
Trainees	14,523	1,113	1,480	15	7.7	101.9	0.9	7.8	1,089 (73.6)
Others^3^	10,250	229	320	11	2.2	31.2	0.2	3.4	151 (47.2)
Total	206,711	13,110	17,506	805	6.3	84.7	10.1	4.6	12,856 (73.4)

^1^Logistic workers refer to those who deal with medical wastes.

^2^SI rates (% HCWs) between different HCW groups were statistically significant (*p* < 0.003125) except those between nurses and trainees as determined by pairwise comparisons.

^3^Others refer to physical therapists, midwives, anesthetists, medical imaging technicians and cleaners for endoscopy, etc.

^4^The reporting rate of trainees was higher than that of doctors and nurses (*p* < 0.003125), while the remaining pairwise comparisons for the reporting rate were not statistically significant (*p* > 0.003125).

**Table 2:** SI rates (incidents 1,000 HCWs per month) stratified by hospital types.

Group^1^	Tertiary hospitals			Secondary hospitals		
	Number surveyed	SI incidents	SI rate, 1,000 HCWs per month	Number surveyed	SI incidents	SI rate, 1,000 HCWs per month
**Doctors**	**37**,**350**	**2**,**787**	**74.6**	**21**,**416**	**1**,**452**	**67.8**
Nurses	61,880	6,482	104.8	36,238	3,713	102.5
**Technicians**	**6**,**027**	**277**	**46.0**	**3**,**909**	**141**	**36.1**
Logistic workers^2^	10,248	567	55.3	4,870	287	58.9
Trainees	9,819	1,015	103.4	4,704	465	98.9
**Others**^3^	**7**,**265**	**190**	**26.2**	**2**,**985**	**130**	**43.6**
Total	132,589	11,318	85.4	74,122	6,188	83.5

^1^Parameters with statistical significance (*p* < 0.05, determined using Chi square test) are highlighted in bold.

^2^Logistic workers refer to those who deal with medical wastes.

^3^Others refer to physical therapists, midwives, anesthetists, medical imaging technicians and cleaners for endoscopy, etc.

**Table 3:** Locations of the occurrence of SIs, case (%^1^)^2^.

HCW group	Patient rooms	Operating rooms	ICU	Outpatients	Emergence	CSSD^3^	Dialysis unit	Lab	Waste storage	Others^5^	NA^6^	Total
Doctors	556 (13.1)	2,620 (61.8)	71 (1.7)	435 (10.3)	130 (3.1)	5 (0.1)	3 (0.1)	13 (0.3)	11 (0.3)	193 (4.6)	202 (4.8)	4,239
Nurses	5,651 (55.4)	835 (8.2)	611 (6.0)	1,112 (10.9)	307 (3.0)	192 (1.9)	75 (0.7)	25 (0.2)	176 (1.7)	659 (6.5)	552 (5.4)	10,195
Technicians	9 (2.2)	4 (1.0)	0 (0.0)	146 (34.9)	3 (0.7)	0 (0.0)	0 (0.0)	146 (34.9)	2 (0.5)	105 (25.1)	3 (0.7)	418
Logistic workers^4^	246 (28.8)	39 (4.6)	35 (4.1)	94 (11.0)	31 (3.6)	49 (5.7)	5 (0.6)	29 (3.4)	181 (21.2)	69 (8.1)	76 (8.9)	854
Trainees	884 (59.7)	164 (11.1)	32 (2.2)	159 (10.7)	65 (4.4)	6 (0.4)	4 (0.3)	3 (0.2)	18 (1.2)	80 (5.4)	65 (4.4)	1,480
Other^5^	45 (14.1)	22 (6.9)	3 (0.9)	35 (10.9)	5 (1.6)	40 (12.5)	0 (0.0)	22 (6.9)	3 (0.9)	91 (28.4)	54 (16.9)	320

^1^%, proportion of SIs ocurred in each location in all SIs for a certain HCW group.

^2^The differences of SIs distribution for HCW groups in different locations were statistically significant (*p* < 0.05, determined using Fisher’s exact test).

^3^CSSD, central sterilization service department.

^4^Logistic workers refer to those who deal with medical wastes.

^5^Others refer to physical therapists, midwives, anesthetists, medical imaging technicians and cleaners for endoscopy, etc.

^6^NA, information is not available.

**Table 4:** Medical procedures leading to SIs, cases (%^1^)^2^.

Medical procedures	Doctors	Nurses	Technicians	Logistic workers^3^	Trainees	Others^4^
Recapping	393 (9.3)	1,087 (10.7)	32 (7.7)	19 (2.2)	233 (15.7)	12 (3.8)
Drawing venous/arterial blood	84 (2.0)	577 (5.7)	95 (22.7)	5 (0.6)	77 (5.2)	13 (4.1)
Intravenous/Intramuscular injection	137 (3.2)	848 (8.3)	4 (1.0)	9 (1.1)	116 (7.8)	8 (2.5)
Preparing fluid infusions	68 (1.6)	1,202 (11.8)	0 (0.0)	4 (0.5)	177 (12.0)	13 (4.1)
Removing venous infusion needles	46 (1.1)	1,579 (15.5)	12 (2.9)	11 (1.3)	253 (17.1)	5 (1.6)
Bruising	135 (3.2)	177 (1.7)	1 (0.2)	0 (0.0)	12 (0.8)	3 (0.9)
Surgical suturing/scalpel	1,818 (42.9)	415 (4.1)	21 (5.0)	5 (0.6)	101 (6.8)	11 (3.4)
Collecting surgical instruments	167 (3.9)	349 (3.4)	4 (1.0)	53 (6.2)	26 (1.8)	35 (10.9)
Treating medical waste	223 (5.3)	1,709 (16.8)	41 (9.8)	605 (70.8)	208 (14.1)	19 (5.9)
Delivering needles and instruments	291 (6.9)	243 (2.4)	9 (2.2)	4 (0.5)	32 (2.2)	2 (0.6)
Adding new doses for infusion	92 (2.2)	762 (7.5)	4 (1.0)	0 (0.0)	74 (5.0)	16 (5.0)
Others procedures	579 (13.7)	696 (6.8)	191 (45.7)	66 (7.7)	107 (7.2)	129 (40.3)
NA^5^	206 (4.9)	551 (5.4)	4 (1.0)	73 (8.5)	64 (4.3)	54 (16.9)
Total	4,239	10,195	418	854	1,480	320

^1^%, proportion of SIs caused by each procedure in all SIs for a certain HCW group.

^2^The differences of SIs distribution for HCW groups in different medical procedures were statistically significant (*p* < 0.05, determined using Fisher’s exact test).

^3^Logistic workers refer to those who deal with medical wastes.

^4^Others refer to physical therapists, midwives, anesthetists, medical imaging technicians and cleaners for endoscopy, etc.

^5^NA, information is not available.
